# Cross-Species and Cross-Polymorph Seeding of Lysozyme Amyloid Reveals a Dominant Polymorph

**DOI:** 10.3389/fmolb.2020.00206

**Published:** 2020-08-14

**Authors:** Lida Rahimi Araghi, Derek R. Dee

**Affiliations:** ^1^Department of Food Science and Technology, University of Georgia, Athens, GA, United States; ^2^Faculty of Land and Food Systems, The University of British Columbia, Vancouver, BC, Canada

**Keywords:** protein aggregation, amyloid strains, heterologous seeding, species barrier, amyloid growth kinetics, polymorphism, lysozyme

## Abstract

The ability to self-propagate is one of the most intriguing characteristics of amyloid fibrils, and is a feature of great interest both to stopping unwanted pathological amyloid, and for engineering functional amyloid as a useful nanomaterial. The sequence and structural tolerances for amyloid seeding are not well understood, particularly concerning the propagation of distinct fibril morphologies (polymorphs) across species. This study examined the seeding and cross-seeding reactions between two unique fibril polymorphs, one long and flexible (formed at pH 2) and the other short and rigid (formed at pH 6.3), of human lysozyme and hen egg-white lysozyme. Both polymorphs could cross-seed aggregation across species, but this reaction was markedly reduced under physiological conditions. For both species, the pH 6.3 fibril polymorph was dominant, seeding fibril growth with a faster growth rate constant at pH 2 than the pH 2 polymorph. Based on fibrillation kinetics and fibril morphology, we found that the pH 2 polymorph was not able to faithfully replicate itself at pH 6.3. These results show that two distinct amyloid polymorphs are both capable of heterologous seeding across two species (human and hen) of lysozyme, but that the pH 6.3 polymorph is favored, regardless of the species, likely due to a lower energy barrier, or faster configurational diffusion, to accessing this particular misfolded form. These findings contribute to our better understanding of amyloid strain propagation across species barriers.

## Introduction

Understanding the structure and assembly mechanisms of amyloid fibrils is of great interest owing to their broad importance. Amyloid fibrils are central to numerous pathologies (Chiti and Dobson, 2017), are increasingly being linked with native biological functions (Fowler et al., 2007; Maji et al., 2009; Liu et al., 2017), and are likely to find applications as biomaterials and devices (Knowles and Mezzenga, 2016). Much remains to be understood about how proteins change their folded structure, self-assemble into protofilaments, and how these (typically 2–6) further combine to form mature fibrils (Adamcik and Mezzenga, 2018).

A given protein can be induced to form distinct amyloid fibril strains, also termed *polymorphs*, that can differ in terms of core structure, morphology, stability, and cytotoxicity (Tycko, 2015; Adamcik and Mezzenga, 2018). Fibril polymorphs have been characterized for several proteins, including human lysozyme (HLZ) (Mossuto et al., 2010), hen egg-white lysozyme (HEWL) (Lara et al., 2011), insulin (Sneideris et al., 2015), α-synuclein (Bousset et al., 2013), serum albumin (Usov et al., 2013), Aβ_1__–__40_ (Petkova et al., 2005), and prion protein (Cobb et al., 2014). Amyloids obtained *in vivo* were found to be polymorphic (Annamalai et al., 2016) and amyloid polymorphism may underlie differences in pathological phenotypes (Peelaerts et al., 2015). Selection between polymorphs may be kinetically controlled, with differences in nucleation barriers determining which nuclei are dominant under a given set of conditions, leading to one polymorph being dominant (Pellarin et al., 2010). Fibril polymorphism is highly sensitive to environmental or chemical effects; this is perhaps best exemplified by the well-characterized Aβ_1__–__40_ peptide that forms distinct polymorphs in response to shaking (striated ribbon fibrils) or not shaking (twisted ribbon fibrils) (Tycko, 2015). The arrangement of water molecules around oligomers and protofilaments during fibrillation could also affect polymorphism (Stephens and Kaminski Schierle, 2019).

In the nucleation-polymerization mechanism (Jarrett and Lansbury, 1993), fibrillation begins with nuclei formation, which strongly affects the lag-phase in the sigmoidal kinetics of fibril production. The lag-phase can be shortened by addition of pre-formed fibrils that act as “seeds.” A protein may also be cross-seeded (Morales et al., 2013) using fibril seeds derived from a similar protein that differs in sequence (e.g., mutants, or from a different species), or using a completely unrelated protein. Cross-seeding may also refer to when fibrils grown under one set of conditions are used as seeds under a different set of conditions (e.g., polymorphic cross-seeding). Cross-seeding is relevant to species barriers in prion diseases (Collinge and Clarke, 2007), to co-aggregation of different proteins in neurodegenerative disease (Hashimoto et al., 2019), and should also be considered when assessing the safety of fibrils engineered for human use. Owing to their novel properties, there is interest in engineering amyloid fibrils for use in materials, devices (Knowles and Mezzenga, 2016), and food (Loveday et al., 2009; Mohammadian and Madadlou, 2018). For applications that may result in human exposure to engineered fibrils, it is prudent to examine their safety (Lasse et al., 2016). One concern is whether fibrils derived from other species may cross-seed amyloid formation of human proteins (Friedland and Chapman, 2017). Cross-seeding is generally inefficient for proteins of low sequence similarity (Krebs et al., 2004; Wright et al., 2005), although, there is evidence of cross-seeding between dissimilar proteins such as amyloid-β and human islet amyloid polypeptide, with 25% sequence identity (Zhang et al., 2015), and curli protein and prostate acid phosphatase (Hartman et al., 2013).

Lysozyme is a well studied model of amyloid formation and numerous methods are known to induce lysozyme fibrillogenesis (Trexler and Nilsson, 2007; Swaminathan et al., 2011). Lysozyme fibrillation can be induced using pH 2 and elevated temperatures (37–90°C), which results in extensive (Frare et al., 2004; Lara et al., 2011) to limited (Arnaudov and de Vries, 2005; Frare et al., 2006) or no (Krebs et al., 2000; Mossuto et al., 2010) hydrolysis of the fibrillar protein, depending on the particular conditions. Alternatively, a less well-characterized method is to use 3 M guanidine hydrochloride (GdnHCl) at pH 6.3 and 45–50°C (Vernaglia et al., 2004) or 3 M GdnHCl at pH 7 and 57°C (Sulatskaya et al., 2017). Previous studies looked at various seeding and cross-seeding reactions of lysozyme fibrils (Krebs et al., 2000, 2004; Vernaglia et al., 2004; Buell et al., 2011; Mulaj et al., 2014). The efficiency of cross-seeding HEWL decreased with the sequence identity of the seed protein: turkey (95% identity) and human (60% identity) lysozyme seeds were equally (turkey) or intermediately (human) effective as HEWL seeds, while α-lactalbumin (36% identity) and bovine insulin (no identity) fibrils were ineffective as seeds (Krebs et al., 2004). In turn, HEWL fibrils were able to seed fibrillation of turkey lysozyme but not human α-lactalbumin (Krebs et al., 2004).

Here, we examined seeding across both species (HEWL and HLZ) and fibril polymorphs, to examine their influence on nucleation barriers and fibril growth. We compared two polymorphs, one formed at pH 2 and one formed at pH 6.3 with 3 M GdnHCl. Based on previous studies (Frare et al., 2004; Vernaglia et al., 2004), we anticipated that fibrils grown under these conditions would differ in terms of morphology (length, stiffness, protofilament assembly) and molecular structure, with hydrolysis likely to occur at pH 2 giving rise to a unique surface chemistry. We show that the pH 2 and pH 6.3 fibril polymorphs of lysozyme differ greatly in their morphology and cross-seeding behavior, with the pH 6.3 polymorph propagating more quickly even at low pH and absent of chemical denaturant. The fibrillar proteins are extensively hydrolyzed at pH 2, but remain intact at pH 6.3. The fibrils formed at pH 6.3 undergo cleavage at pH 2, yet the fibrils remain intact and able to propagate their distinct morphology.

## Materials and Methods

### Materials

Hen egg-white lysozyme (L6876), HLZ (L1667), porcine pepsin (P6887), and Thioflavin T (ThT, T3516), were purchased from Sigma-Aldrich (United States). GdnHCl, was purchased from Fisher (United States). Uranyl acetate and 0.5% formvar solution were purchased from Electron Microscopy (United States). All buffers and solutions were prepared using ultra-pure water (Purelab Ultra, ELGA, United Kingdom).

### Preparation of Fibrils and Seeds

Lysozyme solutions were prepared by mass, centrifuged at 7,800 rcf for 20 min and filtered (0.22 μm) to remove any insoluble protein. Protein concentration was determined by absorbance at 280 nm, measured using a NanoDrop One UV-Vis spectrophotometer (Thermo Scientific, United States), using extinction coefficients of 37,970 and 36,940 M^–1^cm^–1^ for HEWL and HLZ, respectively.

Fibrillation was induced by incubating 1 mM protein solutions, with constant shaking at 300 rpm, in either 50 mM Glycine-HCl, pH 2.0, at 65°C, or in 20 mM potassium phosphate, pH 6.3, containing 3 M GdnHCl, at 50°C. The GdnHCl concentration was determined using refractive index (Pace, 1986). The pH 2.0 samples were incubated for 7 days, and the pH 6.3 samples were incubated for 3 days. Mature fibrils were sonicated at room temperature using a Vibra-Cell VC-50 ultrasonic processor fitted with a flat 2 mm titanium microtip probe (Sonics & Materials Inc., United States). The instrument was operated at 20 kHz frequency and the power was set to 40% ultrasonic amplitude. Samples were placed in an ice bath and were sonicated for three 10 s pulses, with a 5 s rest in between each pulse.

### Fibrillation Kinetics

Experiments were conducted using black, clear-bottom, 96-well microplates (Greiner Bio-One, United States). Fibril seeds (0.1 mg/ml final concentration) were added to freshly prepared native protein solutions (2 mg/ml) in a total volume of 200 μL. 20 μM ThT was included for fluorescence measurements. The microplates were sealed with a black plastic film (Perkin Elmer, United States), incubated at 45°C with 20 s of shaking every 10 min. Fluorescence was measured using a SpectraMax Gemini EM spectrofluorophotometer (Molecular Devices, United States), in bottom-reading mode with excitation at 440 nm and emission at 486 nm. The resulting traces of fluorescence intensity (*I*) over time (*t*) were fit using the equation (Arosio et al., 2015):

(1) $$I=I_{0}+A/\left(1+\exp\left(-k\,\left(t-t_{0.5}\right)\right)\right)$$

where *I*_0_ is the pre-transition baseline, *A* is the transition amplitude, *k* is the apparent rate constant for fibril growth, and *t*_0.5_ is the transition half-time. The lag-time was derived from *t*_lag_ = *t*_0.5_−1/2*k*.

### TEM

Samples for TEM were diluted in MilliQ water (200 μg/mL protein concentration), and 5 μL was placed on 200-mesh copper formvar/carbon-coated grids (Ted Pella, Inc., United States). After 5 min the grids were rinsed three times with 5 μL ultra-pure water, with the excess water removed by blotting with filter paper. Next, 5 μL of 2% (w/v) uranyl acetate solution was placed on the grid, and after 30 s the excess was removed using filter paper and the grids were dried at room temperature. Analysis was performed using a JEOL JEM1011 microscope (JEOL, Inc., United States) at the Georgia Electron Microscopy facility, University of Georgia. Images were analyzed using ImageJ.

### SDS-PAGE

Fibrils were isolated using a 50 kDa spin filter, then diluted with an equivalent volume of tricine sample buffer containing 2% β-mercaptoethanol, and heated at 95°C for 5 min. Samples were run on 16.5% tris-tricine Mini-Protean precast gels (Bio-Rad, United States) at 100 V. Afterward, gels were placed in a fixative solution (40% methanol, and 10% acetic acid) for 30 min, stained for 1 h (0.025% w/v Coomassie blue G-250, 10% acetic acid), and then destained in 10% acetic acid solution.

### Mass Spectrometry

Fibrils were isolated using a 50 kDa spin filter, then analyzed by MALDI-TOF using a Bruker Autoflex time-of-flight (TOF) mass spectrometer at the Proteomics and Mass Spectrometry facility, University of Georgia.

### Pepsin Treatment

Fibril samples (2 mg/ml) were treated with 0.5 mg/ml porcine pepsin (∼1900 U/ml) at pH 2, 37°C, for 24 h, and then analyzed by ThT fluorescence and TEM. Cross-seeding experiments were also conducted under simulated gastric conditions, with 0.5 mg/ml pepsin, pH 1.2, 34 mM NaCl, 37°C (simulated gastric fluid).

## Results

### Formation of Distinct Fibril Polymorph Seeds

**FIGURE 1:**
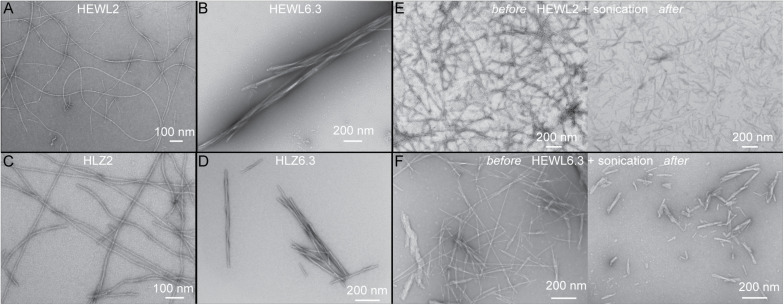
Observation of distinct polymorphs using TEM. Distinct polymorphs of HEWL formed at **(A)** pH 2, and **(B)** pH 6.3. Distinct polymorphs of HLZ formed at **(C)** pH 2, and **(D)** pH 6.3. Panels **(E,F)** show before (left) and after (right) sonication of HEWL mature fibrils to create seeds at pH 2 **(E)** and pH 6.3 **(F)**.

**FIGURE 2:**
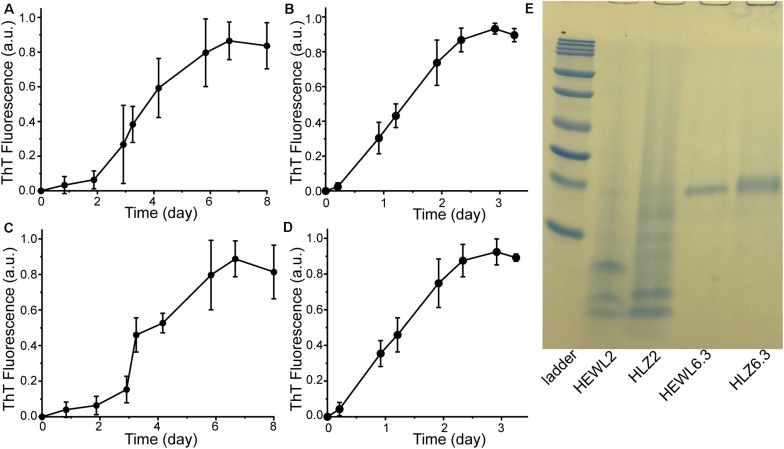
Preparation of mature fibrils to use as seeds. HEWL fibrillation kinetics at **(A)** pH 2 and **(B)** pH 6.3. HLZ fibrillation kinetics at **(C)** pH 2 and **(D)** pH 6.3. All samples were incubated at 65°C with 300 rpm shaking. At each time point, aliquots were taken and combined with ThT for fluorescence measurement. The fluorescence intensity was normalized to the value of the plateau phase in each reaction and data points correspond to the average ± standard deviation of three replicates. **(E)** SDS-PAGE analysis of mature fibrils formed at pH 2 and pH 6.3. Ladder bands are: 10, 15, 20, 25, 37, 50, 75, 100, 150, and 250 kDa.

Hen egg-white lysozyme and HLZ fibril formation was carried out under two different conditions that resulted in two distinct fibril polymorphs (Figure 1). Treating both HEWL and HLZ with pH 2, 65°C and agitation, resulted in the formation of relatively long, flexible fibrils. Treating the proteins with pH 6.3, 3 M GdnHCl, 50°C with agitation resulted in relatively short, straight fibrils, that often displayed a twisted structure and lateral association. Following the kinetics of fibril formation using ThT (Figure 2) revealed that fibrillation was complete by 6 days at pH 2 and by 3 days at pH 6.3, for both HEWL and HLZ. Subsequent analysis by SDS-PAGE (Figure 2E) and MALDI-TOF (see below) revealed that extensive hydrolysis occurred at pH 2, with the pH 2 fibrils comprised of intact and fragmented protein, while the fibrils at pH 6.3 contained full-length protein. Mature fibrils were converted into “seeds” using sonication to break the fibrils down into shorter segments (∼200 nm, Figures 1E,F) generating more fibril ends to serve as the fibril nucleation sites.

### Kinetics of Amyloid Formation

**FIGURE 3:**
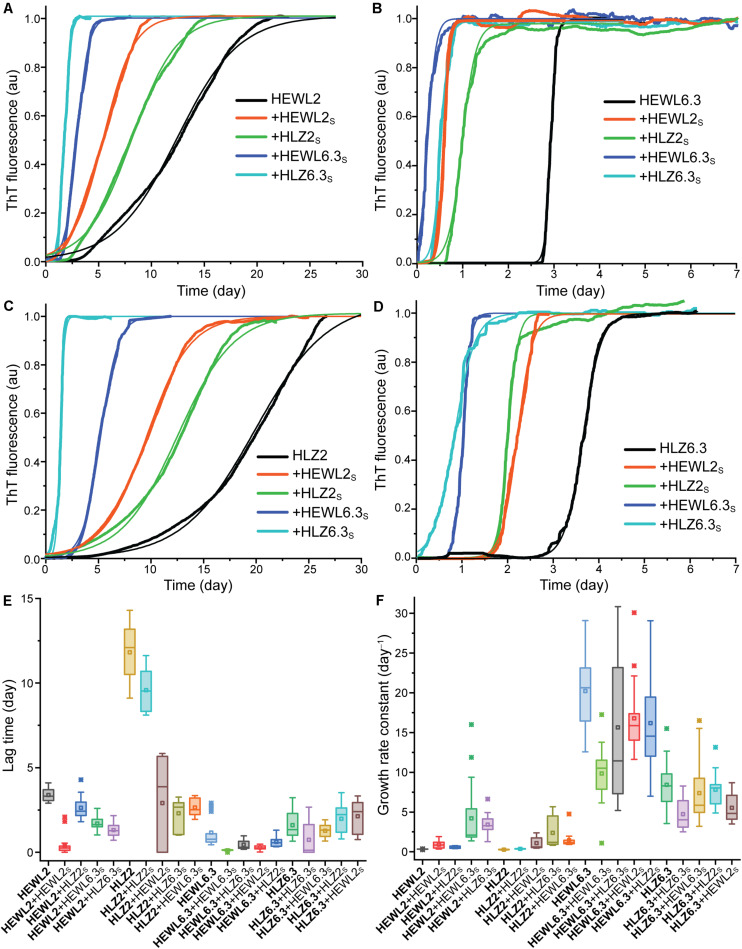
Kinetics of amyloid formation. HEWL at **(A)** pH 2 and **(B)** pH 6.3. HLZ at **(C)** pH 2 and **(D)** pH 6.3. Reactions occurred at 45°C in a microplate reader with intermittent shaking. The seeded reactions are indicated by ‘ + ’ and the seed-type. Representative traces of ThT fluorescence (normalized to the maximum value) are shown, fitted with Eq. 1. Boxplots are shown for **(E)** lag-times and **(F)** growth rate constants, determined by fitting the data to Eq. 1. From left to right, the samples are arranged from unseeded, seeded, cross-seeded, cross-polymorph seeded, to cross-species-cross-polymorph seeded.

Four different seed-types were obtained: HEWL seeds at pH 2 (HEWL2_S_) and pH 6.3 (HEWL6.3_S_), and HLZ seeds at pH 2 (HLZ2_S_) and pH 6.3 (HLZ6.3_S_). Given the large number of samples (four seeds and the unseeded reaction for each condition), a microplate reader-based assay was used to follow the fibrillation kinetics in real-time, where the temperature was limited to 45°C. The kinetics of fibril formation were compared for unseeded (U), seeded (S), cross-species seeded (XS), cross-polymorph seeded (PS), and cross-species-cross-polymorph seeded (XSPS) reactions using ThT fluorescence (Figure 3). The ThT data were fit according to a generic model (Eq. 1), and the fit results are given in Table 1. A number of different effects were observed. *Polymorph effect (pH 2 vs. 6.3)* Fibril formation under both conditions, at pH 2 and pH 6.3 with 3 M GdnHCl, displayed the characteristic sigmoidal growth kinetics with an extended lag-phase followed by an exponential growth and then plateau in the ThT fluorescence intensity (Figure 3). In the absence of seeds, fibrillation at pH 6.3 proceeded with much faster growth rates and shorter lag-times than at pH 2. *Species effect (HEWL vs. HLZ)* Compared to HLZ, fibrillation of HEWL was characterized by a much shorter lag-time yet similar growth rate at pH 2; at pH 6.3, their lag-times were similar yet HEWL had double the growth rate. *Seeding effect* As a nucleation-dependent process, fibrillation typically involves a lag-phase during which sufficient nucleation sites form. This lag-phase can be reduced or eliminated by adding pre-formed fibrils to act as seeds. Here, HEWL2_S_ and HEWL6.3_S_ decreased the fibrillation lag-time by a factor of ∼10, while affecting the growth rate by a factor of only two-three (Table 1). Conversely, HLZ6.3_S_ moderately shifted the lag-time lower (two-fold), while HLZ2_S_ was essentially ineffective as a seed (less than two-fold reduction in lag-time). *XS effect* HLZ2_S_ was also ineffective at cross-seeding HEWL. Conversely, HEWL2_S_ cross-seeded HLZ, decreasing the lag-time and increasing the growth rate by factors of four. No cross-seeding effect was observed for HEWL6.3_S_, while HLZ6.3_S_ did lower the lag-time for HEWL6.3 by nearly three-fold. *PS effect* Perhaps the most striking effect on fibrillation kinetics was observed when using the pH 6.3 seeds at pH 2. HEWL6.3_S_ marginally reduced the lag-time (factor of two) but increased the growth rate constant 13-fold. HLZ6.3_S_ reduced the lag-time by a factor of five and increased the growth rate constant nine-fold. In contrast, HEWL2_S_ and HLZ2_S_ had no effect on the growth rate at pH 6.3, although HEWL2_S_ did reduce the lag-time by a factor of four. *XSPS effect* The polymorph effect was also dominant when seeding across species. HEWL6.3_S_ nucleated the fibrillation of HLZ at pH 2 with a five-fold reduced lag-time and a five-fold increased growth rate. HLZ6.3_S_ seeded HEWL2 with nearly a three-fold lower lag-time and a 10-fold higher growth rate. By comparison, the pH 2 seeds, HEWL2_S_ and HLZ2_S_, had little effect on fibrillation of HLZ and HEWL, respectively, at pH 6.3.

**TABLE 1:** Kinetic parameters of fibril formation.

**Reaction Type**	**Sample**	**Seed**	**Lag-time (day)**	**Factor decrease**	**Half-time (day)**	**Rate constant (day^–1^)**	**Factor increase**
U	HEWL 2	–	3.380.10		12.170.25	0.330.01	
S	HEWL 2	HEWL2_S_	0.390.12	9	2.900.26	0.910.10	3
XS	HEWL 2	HLZ2_S_	2.630.21	1	6.870.20	0.580.04	2
PS	HEWL 2	HEWL6.3_S_	1.700.09	2	2.850.17	4.210.94	13
XSPS	HEWL 2	HLZ6.3_S_	1.320.10	2.6	2.260.10	3.420.31	10
U	HLZ 2	–	11.820.52		19.530.18	0.270.01	
S	HLZ 2	HLZ2_S_	9.580.30	1	14.610.39	0.370.01	1
XS	HLZ 2	HEWL2_S_	2.911.01	4	5.591.32	1.090.20	4
PS	HLZ 2	HLZ6.3_S_	2.300.21	5	3.990.44	2.380.47	9
XSPS	HLZ 2	HEWL6.3_S_	2.650.12	4	4.540.21	1.450.22	5
U	HEWL 6.3	–	1.160.17		1.290.17	20.220.97	
S	HEWL 6.3	HEWL6.3_S_	0.120.01	10	0.360.02	9.840.81	0.5
XS	HEWL 6.3	HLZ6.3_S_	0.450.06	2.6	0.690.04	15.652.25	0.8
PS	HEWL 6.3	HEWL2_S_	0.280.03	4	0.460.02	16.791.05	0.8
XSPS	HEWL 6.3	HLZ2_S_	0.620.07	2	0.800.07	16.201.46	0.8
U	HLZ 6.3	–	1.610.20		1.970.22	8.430.78	
S	HLZ 6.3	HLZ6.3_S_	0.730.31	2.2	1.360.20	4.720.53	0.6
XS	HLZ 6.3	HEWL6.3_S_	1.270.10	1.3	1.680.13	7.381.07	1
PS	HLZ 6.3	HLZ2_S_	1.980.22	0.8	2.310.22	7.820.50	1
XSPS	HLZ 6.3	HEWL2_S_	2.130.24	0.8	2.600.25	5.530.45	0.7

*Samples correspond to unseeded (U), seeded (S), cross-species seeded (XS), cross-polymorph seeded (PS), and cross-species-cross-polymorph seeded (XSPS) reactions. Data were obtained by fitting individual ThT kinetic traces with Eq. 1 and represent the mean ± SEM of 11–25 replicates.*

Overall, there was a striking effect of species on seeding ability of the fibrils made at pH 2: while the HEWL fibrils made at pH 2 (HEWL2_S_) served as effective seeds, cross-species seeds, and cross-polymorph seeds, the HLZ2_S_ had little observed effect on the fibrillation kinetics under the conditions tested. However, both HEWL and HLZ fibrils made at pH 6.3 served as excellent seeds in the PS and XSPS reactions at pH 2.

### Structure of Seeded Fibrils

**FIGURE 4:**
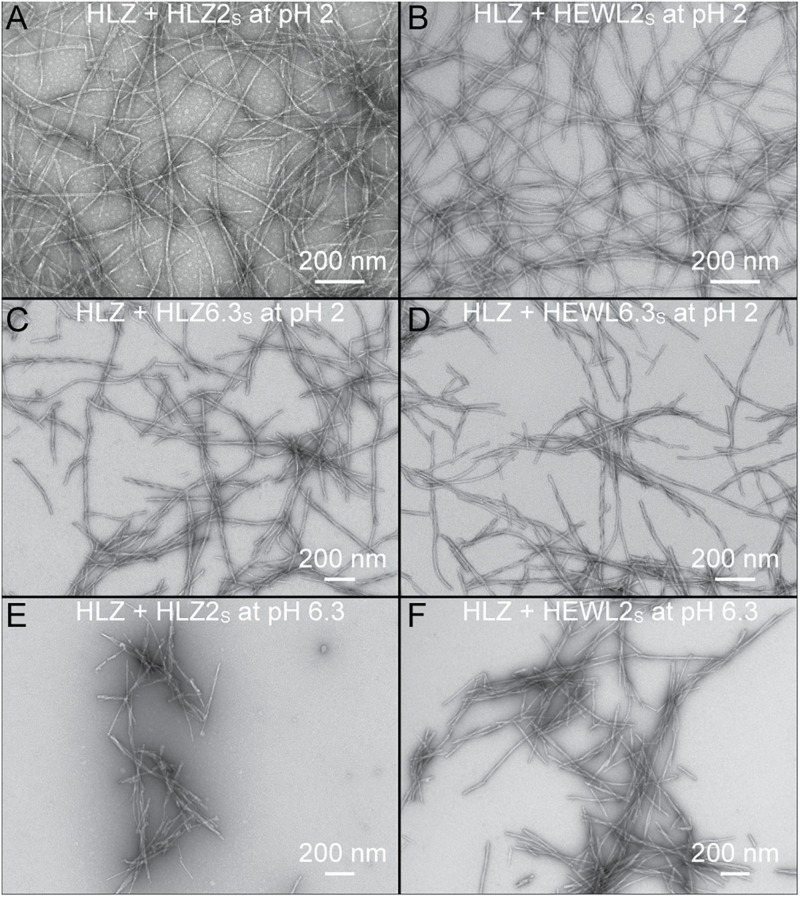
Morphology of HLZ amyloid fibrils produced using various seeds and different conditions. **(A)** HLZ seeded with HLZ2_S_ at pH 2. **(B)** HLZ cross-seeded with HEWL2_S_ at pH 2. **(C)** HLZ cross-polymorph seeded with HLZ6.3_S_ at pH 2. **(D)** HLZ cross-species-cross-polymorph seeded with HEWL6.3_S_ at pH 2. **(E)** HLZ cross-polymorph seeded with HLZ2_S_ at pH 6.3 with 3 M GdnHCl. **(F)** HLZ cross-species-cross-polymorph seeded with HEWL2_S_ at pH 6.3 with 3 M GdnHCl.

Human lysozyme fibrils that resulted from the various types of seeding reactions (S, XS, PS, or XSPS) were analyzed by TEM to determine if the pH 2 and pH 6.3 seeds faithfully propagated their distinct morphology (Figure 4). Monomeric HLZ, at pH 2, seeded with HLZ2_S_ or cross-seeded with HEWL2_S_ produced long, flexible fibrils (Figures 4A,B) that looked identical to those produced during the unseeded reactions (Figures 1A,C). HLZ, at pH 2, cross-polymorph seeded with HLZ6.3_S_ or cross-species-cross-polymorph seeded with HEWL6.3_S_, produced fibrils that were shorter and straighter (more rigid) (Figures 4C,D), being more similar to the unseeded fibrils produced at pH 6.3 (Figures 1B,D). Treating monomeric HLZ, at pH 6.3, with HLZ2_S_ or HEWL2_S_, also produced short, rigid fibrils (Figures 4E,F) similar to the unseeded fibrils at pH 6.3 (Figures 1B,D). These results indicated that the pH 6.3 polymorphs (HLZ6.3_S_ and HEWL6.3_S_) were able to faithfully propagate at pH 2 in the absence of 3 M GdnHCl, while the pH 2 polymorphs were not able to replicate under the pH 6.3 condition. Together with the kinetic results from ThT fluorescence, the TEM structures support that the pH 6.3 polymorph is dominant and favored over the pH 2 polymorph under both conditions of pH 2 and pH 6.3.

### Effect of Low pH and Simulated Gastric Conditions on Fibril Structure

**FIGURE 5:**
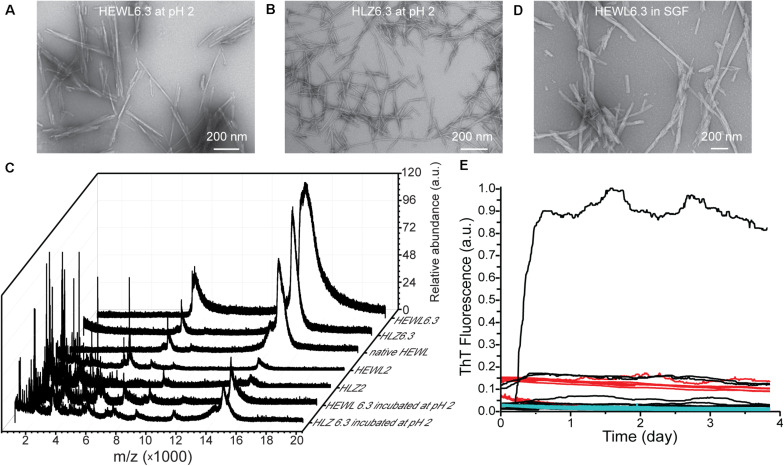
Effect of low pH and simulated gastric fluid on fibril structure and seeding ability. TEM images of HEWL6.3 **(A)** and HLZ6.3 **(B)** after incubation for 24 h at pH 2, 65°C, while shaking at 300 rpm. **(C)** MALDI-TOF spectra for native HLZ and various fibrils. Effect of simulated gastric fluid on HEWL fibril structure **(D)**, and on the ability of HEWL6.3_S_ and HEWL2_S_ to seed HLZ fibrillation **(E)**. Native human lysozyme was incubated with HEWL fibrils in simulated gastric fluid, pH 1.2, 37°C, containing pepsin, over nearly 4 days. Different HEWL fibril seeds were used: HEWL6.3 pre-treated with pepsin (black curves), and HEWL6.3 (red curves) and HEWL2 (cyan curves) sonicated fibrils (*n* = 12 for each seed type).

The pH stability of the pH 6.3 polymorphs was examined by incubating mature HEWL6.3 and HLZ6.3 fibrils at pH 2, 65°C, with shaking at 300 rpm, for 24 h. Analysis by TEM and MALDI-TOF (Figure 5) showed that the fibril morphology remained intact, even though the proteins underwent hydrolysis and the fibrils contained substantial fragmented protein after incubation at pH 2. MALDI-TOF results (Figure 5C) confirmed the SDS results (Figure 2E), indicating that fibrils made at pH 2 and pH 6.3 differed in composition. The pH 2 polymorph consisted of intact and hydrolyzed protein, while the pH 6.3 polymorph contained only intact lysozyme. HEWL and HLZ are 14.3 and 14.7 kDa, respectively, and no bands at higher Mw were observed by SDS-PAGE (Figure 2E), suggesting that fibril formation involved only non-covalent bonds, which were broken down by heating in the presence of SDS (Arnaudov and de Vries, 2005).

Having observed lysozyme cross-seeding at pH 2, 45°C, we next examined whether seeding across species and polymorphs would occur under more physiological conditions, in simulated gastric fluid. HLZ monomers were incubated with various HEWL fibrils (HEWL6.3 pre-treated with pepsin, and HEWL6.3 and HEWL2 sonicated fibrils) in simulated gastric fluid and monitored using ThT fluorescence (Figure 5E). Within a period of 4 days, only one out of 36 samples formed fibrils under simulated gastric conditions, corresponding to one of 12 HLZ samples that was seeded with HEWL6.3 fibrils pre-treated with 0.5 mg/mL pepsin. To examine whether the fibrils were resistant to pepsin treatment, HEWL6.3 fibrils were incubated for 24 h with pepsin (2000 U/ml, pH 2, 37°C). There were no major changes in HEWL6.3 fibril structure after pepsin treatment (Figure 5D), indicating that the fibril core was resistant to pepsin digestion.

## Discussion

### Polymorph Fidelity Under Different Conditions

Lysozyme fibrils were produced under two conditions to yield distinct polymorphs: one at low pH with heating (“pH 2 fibrils”) and the other at pH 6.3 with 3 M GdnHCl (“pH 6.3 fibrils”). The pH 2 fibrils are long, thin, semiflexible, and curved, while the pH 6.3 fibrils are shorter, rigid, twisted, and associate laterally (Figure 1). These results are consistent with previous studies of lysozyme fibrils made under acidic and near-neutral pH conditions (Frare et al., 2004; Krebs et al., 2004; Vernaglia et al., 2004; Mossuto et al., 2010; Mocanu et al., 2014; Sulatskaya et al., 2017).

The unseeded, seeded, and cross-species seeded reactions at pH 2 all resulted in the formation of long, unbranched, flexible fibrils (Figure 4). Conversely, seeding with the pH 6.3 polymorphs at pH 2 resulted in the formation of shorter, more twisted and rigid fibrils, resembling the pH 6.3 polymorphs. This indicates that 6.3 polymorphs were dominant even at pH 2, and the newly formed fibrils preserved the pH 6.3 polymorph fibril structure and morphology. The faithful propagation of polymorphs under different conditions has been observed for other fibrils, including from Aβ_1__–__40_ (Petkova et al., 2005), insulin (Surmacz-Chwedoruk et al., 2012), and α-synuclein (Peelaerts et al., 2015). Such strain fidelity can be explained on the basis of energetic barriers to fibril nucleation, as the barrier to forming a new fibril (even one of the type usually favored under the given conditions) is higher than the barrier to fibril growth; thus, it costs less energy to add a monomer to an existing fibril than to form a completely new fibril (Buell, 2019). What is interesting in this case is that the pH 6.3 fibril polymorphs (HLZ6.3_S_ and HEWL6.3_S_) cross-seeded fibril growth at pH 2 with rate constants nearly 10-fold higher than unseeded fibril growth at pH 2 (Table 1).

It is not immediately clear why the pH 6.3 polymorph grows with a faster rate constant at pH 2 than the pH 2 polymorph. The rate constant for fibril elongation depends both on a diffusive pre-factor and a free energy barrier (i.e., *k* = *k*_0_exp(−Δ*G*^‡^/*RT*). The energetic barrier likely corresponds to a transition state complex formed between the fibril end and the incoming monomer (Buell, 2019). The pre-factor can be taken to arise from internal conformational dynamics of the transition state complex, which was shown to be much slower for protein misfolding than for native folding (Yu et al., 2015; Dee and Woodside, 2016). In this scenario, the pH 6.3 and pH 2 lysozyme polymorphs could differ in terms of their core molecular structure, such that fibril-monomer interactions are more favorable with the pH 6.3 polymorph. While the pH 6.3 fibrils consist of full-length protein at pH 6.3 (Figure 2E), incubation at pH 2 leads to hydrolysis of surface residues leaving behind intact fibrils composed of fragmented protein (Figure 5). Nonetheless, the fibril core remains intact at pH 2, which indicates that the residues forming the fibril core, rather than regions outside the core, are what dictate the seeding and growth kinetics of the pH 6.3 polymorph. This is not necessarily surprising, as assembly of the fibril core would represent the largest energy barrier and also driving force of fibrillation (e.g., through formation of binding contacts and release of water from the buried surfaces). Hydrolysis does not preclude the self-propagation of the pH 6.3 polymorph at pH 2, indicating that the pH 2 and pH 6.3 fibrils can be assembled from the same pool of full-length and truncated protein, although we do not know if they share the same fibril core sequence. The molecular structures of these polymorphs might differ in several ways (Eisenberg and Sawaya, 2017): they could consist of the same core sequence folded in different topologies (“packing polymorphs”); they might consist of different segments (“segmental polymorphs”), or they may arise as a combination of different segments and packing interactions (“combinatorial polymorphs”).

Fibril polymorphism, in general, arises from differences in the structure of the fibril core (molecular polymorphism) and/or differences in fibril morphology due to the protofilament arrangement (morphological or structural polymorphism) (Tycko, 2015; Adamcik and Mezzenga, 2018). Differences in the later can also have a major impact on fibril growth kinetics. For Aβ_1__–__40_ fibrils one polymorph (ribbon-type) was dominant over another (twisted-type) during agitation, owing to it being more susceptible to shear and generating more fibril ends (Qiang et al., 2013). In our case, both polymorphs (pH 2 and pH 6.3) were susceptible to shear via sonication (Figures 1E,F), producing seeds on the order of 200 nm long. The pH 6.3 fibrils are characteristically shorter than the pH 2 fibrils, so it is possible that the pH 6.3 fibrils are more brittle, undergoing breakage during shaking in the plate-reader, resulting in more fibril ends and enhanced fibril growth kinetics (Surmacz-Chwedoruk et al., 2012). An increased fragmentation rate could lead to both shorter lag-times and faster growth kinetics, as fibril growth rate is proportional to the fibril number concentration (Arosio et al., 2015). The pH 6.3 fibrils are characterized by further lateral association (Figures 1–5), which potentially affects the rates of elongation and secondary nucleation, although the details for this are not clear (Arosio et al., 2015).

We also expect to see some effect of net charge on seeding and fibril growth (Buell et al., 2013). The overall faster fibril growth kinetics at pH 6.3 than at pH 2 likely result from electrostatic effects. With a measured *pI* of 11.16, lysozyme is more positively charged (*q* ∼ +17) at pH 2 than at pH 6.3 (*q* ∼ +8) (Kuehner et al., 1999), which could result in less electrostatic repulsion between the growing fibril end and incoming monomer at pH 6.3.

### Cross-Species Seeding of Lysozyme

The kinetics of fibril growth are generally faster for HEWL than HLZ. For the unseeded reactions at pH 2, HEWL has nearly a four-fold shorter lag-time, yet the rate constants are similar. At pH 6.3, the lag-times are similar yet HEWL has double the growth rate constant. This suggests that, compared to HLZ, fibril nuclei formation is faster for HEWL at pH 2 while fibril elongation is faster for HEWL at pH 6.3. Similarly, a previous study found that HEWL aggregates more quickly than HLZ at acidic pH, under slightly different conditions (pH 1.5, 65°C, with stirring), with a shorter lag-time but also a four-fold faster growth rate constant (Chaudhary et al., 2017). This general trend held up across the different seeding and cross-seeding samples examined here, where HEWL aggregation was faster in terms of lag-times and/or rate constants. Although HEWL aggregation was overall faster, trends in the effectiveness of the different fibrils (species and polymorph type) to act as seeds were more nuanced.

The molecular and morphological factors that underly cross-seeding are not well-understood, yet cross-seeding is generally inefficient for proteins of low sequence similarity (Tycko, 2015). This is not an absolute relationship, as similar proteins like Aβ_1__–__40_ and Aβ_1__–__42_ fibrils do not cross-seed efficiently (Lu et al., 2013) while different proteins such as α-synuclein and tau fibrils do cross-seed each other (Giasson et al., 2003). We found that lysozyme from human and hen, with 60% sequence identity, cross-seeded each other under both conditions (pH 2, pH 6.3) with differing efficiencies. HEWL2_S_ reduced the lag-time of HLZ2 by a factor of four, while HLZ2_S_ was a poor seed and cross-seed, leading to only a small drop in the lag-time (less than two-fold reduction) for both human and hen lysozyme at pH 2. This result is consistent with the findings of Krebs et al. (2004), who found that HLZ fibrils prepared at pH 2, 65°C, reduced the lag-time for HEWL from around 2.5 days (unseeded) to 1.5 days (cross-seeded). Conversely, under the pH 6.3 condition, HEWL6.3_S_ had minimal effect on the lag-time of HLZ6.3, while HLZ6.3_S_ reduced the lag-time of HEWL6.3 by nearly three-fold (Table 1).

### Seeding Under Mild (Non-denaturing) Conditions

Previous studies have shown that HEWL fibrils are unable to seed fibril formation under mild or non-denaturing conditions (e.g., without GdnHCl or low pH with high temperature). Fibril seeds made at pH 6.3, 3 M GdnHCl, 50°C, were unable to seed fibril formation at 45°C without 3 M GdnHCl, but did so with denaturant present (Vernaglia et al., 2004). HEWL fibrils made at pH 3 could not seed fibrillation within 48 h at pH 3, 37°C, or within 72 h at pH 7, 37°C (Mulaj et al., 2014). Here, we looked for HEWL6.3_S_ cross-seeding of HLZ under simulated gastric conditions (pepsin, pH 1.2, 34 mM NaCl, 37°C) and found that only one out of 36 samples showed signs of amyloid formation after 4 days (Figure 5E). Such a low propensity for amyloid fibril formation, even in the presence of fibril seeds, might be expected when the solution conditions are well below the equilibrium denaturation mid-point (Buell et al., 2011). The *T*_m_ for HEWL is approximately 45°C at pH 1, and 50°C at pH 2, while the *T*_m_ for HLZ is around 52°C at pH 2 and 80°C at pH 6 (Trexler and Nilsson, 2007). At pH 6.3, 50°C, the denaturant mid-point for HEWL is around 2.5 M GdnHCl, and the protein is 80% denatured at 3 M GdnHCl (Vernaglia et al., 2004). Without sufficient destabilizing conditions, the proteins do not readily aggregate into fibrils. Overly denaturing conditions also disfavor fibrillation (Vernaglia et al., 2004). In the absence of denaturant, pepsin treatment might lead to partial digestion and release of fibril-forming fragments. While HEWL is resistant to pepsin, HLZ is more susceptible (Polverino de Laureto et al., 2002; Frare et al., 2006). Yet, even with pepsin, we observed a low, but non-zero, propensity for fibrillation. These results indicate that, in the absence of more destabilizing conditions, cross-seeding is quite rare. However, these results also lend support to the notion that fibril growth kinetics can be highly sensitive to the particular environmental conditions and the identity of the seeds and monomeric protein.

The issue of cross-seeding is relevant to assessing the safety of engineered amyloid for various technology applications (Knowles and Mezzenga, 2016), particularly in food (Solomon et al., 2007; Raynes et al., 2014; Lasse et al., 2016). While fibrils made from various milk, egg, and legume proteins showed no evidence of cell toxicity (Lasse et al., 2016), further studies are required that address cross-seeding along with cell toxicity. Different polymorphs should be examined, as some may be toxic while others are not (Mossuto et al., 2010). Our results highlight that certain polymorphs, or strains, are more proficient in seeding, even under conditions that differ from which they were formed. This cross-species-cross-polymorph seeding should be considered when assessing the safety of engineered fibrils.

These results further indicate that polymorphism and strain fidelity is a unique feature of fibrils that could be utilized when creating fibril-based materials. Fibrils with desired properties can be formed under one set of conditions (pH, temperature, ionic strength, chaotropes) and then used as seeds to scale production of identical fibrils under a different set of conditions, which may offer downstream advantages.

## Data Availability Statement

The raw data supporting the conclusions of this article will be made available by the authors, without undue reservation.

## Author Contributions

Both authors designed the research, analyzed the data, and wrote the manuscript. LR performed the experiments.

## Conflict of Interest

The authors declare that the research was conducted in the absence of any commercial or financial relationships that could be construed as a potential conflict of interest.
